# Diagnosis and treatment for the early stage of cytomegalovirus infection during hematopoietic stem cell transplantation

**DOI:** 10.3389/fimmu.2022.971156

**Published:** 2022-09-23

**Authors:** Jiaqi Cui, Kui Zhao, Yanling Sun, Ruijuan Wen, Xiangzhong Zhang, Xudong Li, Bing Long

**Affiliations:** Department of Hematology, The Third Affiliated Hospital, Sun Yat-sen University, Guangzhou, China

**Keywords:** diagnosis, treatment, CMV, HSCT, immune recovery

## Abstract

Cytomegalovirus (CMV) infection remains a frequent complication after hematopoietic stem cell transplantation (HSCT) and causes significant morbidity and mortality in transplantation recipients. In this review, we highlight the role of major risk factors that are associated with the incidence of CMV infection. Advances in immunosurveillance may predict CMV infection, allowing early interventions to prevent severe infection. Furthermore, numerous therapeutic strategies against CMV infection after HSCT are summarized. A comprehensive understanding of the current situation of CMV treatment may provide a hint for clinical practice and even promote the development of novel strategies for precision medicine.

## 1 Introduction

Allogeneic hematopoietic stem cell transplantation (allo-HSCT) is considered to be the strongest curative treatment for malignant hematologic diseases. It begins with a conditioning regimen that destroys cancer cells and the immune system, then allows the recipient to accept an immune system-reconstituting infusion of donor stem cells. Before the hematopoiesis and immune reconstitution, allo-HSCT recipients are susceptible to infection by bacteria, fungi, or viruses, which affect the outcome after allo-HSCT. Among those pathogens, cytomegalovirus (CMV) reactivation is considered one of the most serious challenges during HSCT.

CMV, classified as the beta-human-herpesvirus type 5 (HHV-5), is prevalent globally, with seroprevalences ranging from 30% to over 90% according to age and geographical factors (1–3). Primary CMV infection typically progresses to latency in human epithelial tissue, polymorphonuclear cells, myeloid progenitors, and T lymphocytes, for it is normally inhibited by the host’s immune system. In those who have received HSCT, latent CMV is often reactivated in the early stages of immune recovery, usually within 3-4 months after HSCT (4). Early CMV infection before day 100 after HSCT leads to numerous end-organ diseases, increases the risk of acute or chronic graft-versus-host disease (GVHD), enhances invasive fungal infection, contributes to graft failure and fatal outcomes (5, 6). In post-transplant patients, early infection was also mentioned to be associated with increased non-relapse mortality, which appears to be linked to complex effects on the post-transplant CMV-specific T cell recovery (7–9). Thus, in this review, we focus on major risk factors, diagnosis, and management of early CMV infection after HSCT.

## 2 Risk factors for CMV infection

As suggested by current studies, there are multiple risk factors (**Table 1**) that predispose a patient to CMV infection, such as the states of both donors and recipients, and the strategies during HSCT. Among all the risk factors mentioned above, four of them are considered major: CMV serostatus, acute or chronic GVHD, type of donor, and *in-vivo/ex-vivo* T cell depletion (10, 11).

**Table 1:** Risk factors for CMV infection in HSCT.

**Recipients**
CMV serostatus (R^+^)
age (elder)
**Donor**
CMV serostatus and match (mismatch)
human leukocyte antigen (HLA) match (mismatch)
type of donor (unrelated donor)
age (elder)
**Pre-transplant**
Conditioning regimen (Myeloablative conditioning regimen, MAC)
T-cell depletion (high dose of ATG/ATLG)
**Post-transplant**
prevention for GVHD (use of immune suppressors)
occurrence GVHD
treatment of GVHD (use of steroids and immune suppressors) immune reconstitution (recovery of CMV CTLs)

### 2.1 CMV donor and recipient serology

In allo-HSCT recipients, the most important risk factor for CMV infection is the CMV serological status of both donors and recipients (12). CMV seropositive recipients (R+) have the highest incidence of reactivation, especially when they were transplanted from seronegative donors (D^−^) (13–15). The D^−^/R^−^ have the lowest risk of CMV infection (16). In D^+^/R^+^ patients, reactivation may derive from endogenous latent CMV in the R^+^ and/or from latently infected cells transferred within the D^+^ stem cell graft. D^−^/R^+^ experience the incidence of invasive CMV disease, which could be a consequence of delayed CMV-specific immune recovery owing to the lack of pre-existing CMV-specific memory T cells in the graft (14, 15). The CMV-specific memory T cells transferred with D^+^ grafts facilitate antigen-driven amplification in the recipient early after transplantation and contribute to early control of the reactivation (14, 15). As a result, whenever possible, attempts are made to match serostatus in donors and recipients in current clinical practice. However, there are debates on the benefits of transferred CMV-specific T cell (17, 18). Some believed that the benefit is very limited for the number of CMV-specific memory T cells transferred is small. Moreover, HSCT recipients are routinely treated with immunosuppressive agents to prevent GVHD, which will also inhibit the function of CMV-specific T cells.

### 2.2 GVHD

The incidence of GVHD increases the risk of CMV infection, especially with its treatment by steroids (19, 20). Similar to immunosuppressive drugs, steroids suppress the immune system by inhibiting CMV-specific T cell activation. In return, CMV infection may also increase the risk of GVHD. As reported, CMV-infected cells promote the production of IL-6 and lead to GVHD (21, 22). Years ago,​ Styczynski’s project showed that CMV infection occurred nearly twice as much in patients with acute GVHD as in those without acute GVHD [*P<0.0001*, 60.1% (885/1472) *vs* 32.1% (892/2780)] (23). Moreover, enhanced transplant-related mortality and decreased overall survival were reported in those R^+^ recipients with acute GVHD (19).

### 2.3 Type of donor

Recipients of transplants from different types of donors have been analyzed in multiple studies, including match-related donors (MRD), match-unrelated donors (MUD), haploids, and umbilical cord blood (UCB). Accordingly, CMV infection occurred in less than 40% of MRD transplant recipients (24–28), while in more than half of MUD (24, 25, 27) and haploid cases (25–28), respectively. Clearly, the risk of CMV infection was higher in MUD and haploid transplants than that in MRD (OR 1.96, 95% 1.76-2.20, *P<0.0001*). One of the reasons is that recipients from HLA mismatched donors are prone to develop GVHD. Alloimmune responses mediated by GVHD impair thymopoiesis, which delays the reconstitution of CMV-specific T cells (29). Moreover, recipients of UCB transplantation have the highest incidence of CMV infection, with an infection rate of 74.5% (246/330) (30–33). The T cells in cord blood grafts are naive and have impaired functional recovery, which contributes to prolonged reconstitution of antigen-specific immunity and increased risks of viral infection (34). Yet recipients from mismatch-unrelated donors (MMUD) were seldom reported.

### *2.4 In-vivo/ex-vivo* T cell depletion (TCD)

To prevent severe acute and chronic GVHD (aGVHD and cGVHD), both *in-vivo and ex-vivo TCD* are used as part of a myeloablative conditioning regimen prior to allo-HSCT in malignant diseases. However, these approaches are associated with delayed immune reconstitution and an increased risk of infection (26). Compared to *in-vivo* TCD, traditional *ex-vivo* TCD experiences a higher incidence of infectious complications, and relapse (35). Thus in the current clinical practice, *in-vivo* TCD, such as post-transplant cyclophosphamide (PTCy) and anti-thymocyte (ATG), is the most frequently used method to prevent GVHD. With the beneficial effect on severe aGVHD and cGVHD, PTCy resulted in a higher incidence of CMV infection than other *in-vivo* TCD approaches (26). ATG is generally considered to deplete T cells effectively and allow great expansion of regulatory T cells, which release inflammatory factors (such as IL-10 and TGF-β) to inhibit GVHD (36). Compared with patients not receiving ATG, this TCD was reported to slow down the recipient’s reconstitution of CD4^+^ and CD8^+^ T cells in the peripheral blood (37, 38). Different ATG regimens are applied in current studies, and CMV infections vary, but many support that a lower dose decreases the risk of CMV infection (39–41).

In conclusion, CMV infection should be regarded as a consequence of the single or multiple risk factors mentioned above. Optimization of these factors helps to achieve a lower viral activation rate.

## 3 Post-HSCT immune recovery after CMV infection

**Figure 1:**
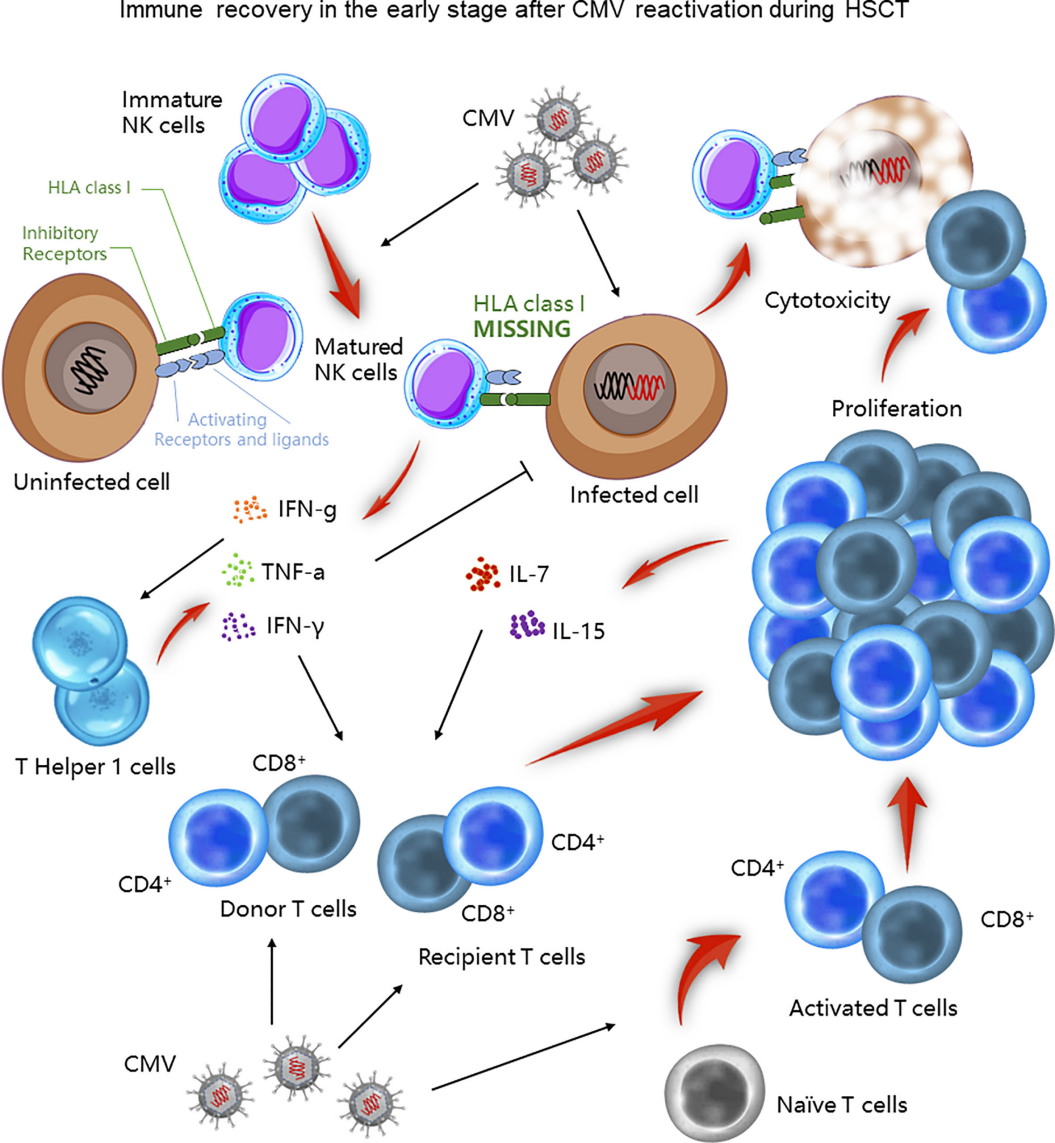
Immune recovery in the early stage after CMV infection during HSCT. During the first 100 days after HSCT, NK and T cells play a crucial role in controlling CMV infection. Matured NK cells directly lyse CMV infected cells or indirectly facilitate Th1 responses to control CMV infection. T cells also exert their cytotoxic function through both existing and newly generated populations.

After the transplantation, neutrophils are the first cell line to reconstitute, followed by monocytes, natural killer (NK) cells, and T cells by day 100, and B cells take 1 to 2 years (42–44). During the first 100 days after HSCT, NK cells and T cells are reported to be the major immune cells that protect the recipient from CMV infection (**Figure 1**). NK cells are the first lymphocytes to recover in HSCT recipients, but are functionally impaired. When CMV reactivates, it stimulates and expands a distinctive NK cell population that is able to secrete cytokines and be cytotoxic. These activated NK cells express numerous activating and inhibitory receptors on their surface, matching with the corresponding ligands on healthy cells (45). One group of the inhibitory receptors, namely killer cell immunoglobulin-like receptors (KIRs), specifically recognizes class I HLA, an inhibitory ligand expressed on normal cells (45, 46). The balance of receptors and ligands is essential to keep healthy cells alive. However, class I HLA is down-regulated in CMV-infected cells, breaking down the balance. Both the undermined inhibitory effect and the superiority of activating receptors-ligands signals initiate cytotoxicity of activated NK cells (45, 47). These NK cells also secrete cytokines, including tumor necrosis factor-a (TNF-a) and interferon-g (IFN-g), which likely contribute to the early control of the CMV infection (45). Moreover, activated NK cells provide an early source of IFN-g, promoting T helper cell type 1 (Th1) responses, which further enhances CD4^+^ T cell against CMV infection by secreting IFN-γ and TNF-α (48).

On the other hand, CMV-specific CD4^+^ and CD8^+^ T cells also play an important role against CMV infection. The early post‐transplant period is characterized by the peripheral expansion of either donor T cells in the graft or recipient T cells that survived conditioning. Their memory feature urges them to react and proliferate rapidly soon after CMV infection, thereby killing the infected cells. In response to lymphopenia, proliferated T cells also secrete interleukin (IL) -7 and IL-15 to promote their reconstitution. Yet, these T cells are with restricted T cell receptor (TCR) repertoire, limited antigen specificity, and are alloreactive, which is capable to cause GVHD (29). In contrast, complete reconstitution of the TCR relies on bone-marrow-derived and thymus-matured naive T cells (29). Compared to the early period, this broadens the TCR diversity of a *de novo* group of T cells. Intriguingly, this virus was reported to directly infiltrate the thymic epithelium, activating cytotoxic T-cell responses toward the thymus, and therefore impairing this complete immune reconstitution (49).

## 4 Early detection for CMV infection

To include and benefit as many patients as possible, particularly for those with inevitable high risks, monitoring viral DNA is believed to be another crucial approach. Rapid confirmation of CMV infection will alert the practitioners, and clinical strategies will be made in time to reduce CMV-related mortality early after transplantation. With the development of technology, the ways of monitoring CMV status vary from time to time.

### 4.1 Traditional methods for clinical diagnosis

Over the years, several diagnostic methods have been developed to detect CMV infection. The most commonly used methods are detections of the CMV phosphoprotein (pp65) antigen by antigenemia assay and DNA viral load using CMV Quantitative Nucleic Acid Test (QNAT) (50).

#### 4.1.1 CMV antigen

Detecting this viral structural protein (51) was once a feasible way to diagnose CMV infection for its operational and economic advantages. However, it has been gradually replaced by CMV QNAT, another detective approach that aims specifically at CMV DNA.

#### 4.1.2 CMV QNAT

The application of CMV QNAT has been increased in recent years given its high sensitivity and quantification of viral load that conveys important prognostic information of patients (52, 53). Yet, there are some limitations. For instance, the results vary depending on the specimen types. Most CMV DNA in plasma consists of small fragments, which increases the measured values and causes bias. Studies suggested that most viral load values in plasma are about 10-fold higher than that in the whole blood (52), for both cell-free and intracellular virus DNA can be detected. Hence, a fixed sample type is recommended for serial viral load monitoring. Moreover, there is no universal standard for viral load, due to the lack of standardization among various commercial and laboratory-developed assays. Although the W.H.O. expert committee established an International Standard (IS) for CMV QNAT in October 2010, some variability in the test results still remains (54). Therefore, most transplant centers have to establish their own threshold for preemptive therapy (55, 56).

### 4.2 Novel methods for prediction

The pathogenesis of CMV infection is complex, and it interacts with the immune system in many ways, mainly through cellular immunity and humoral immunity. As recently reported, cytokines produced by CMV-specific CD4^+^ and CD8^+^ T helper cells, as well as CMV-specific antibodies produced by the humoral immune response can be used to predict and determine the CMV infection (57–60).

#### 4.2.1 CMV-specific antibodies

Recipient CMV seropositivity is a known risk factor for CMV infection after transplantation, some studies have reported that CMV-IgG titers before transplantation can predict the risk of CMV infection (57, 58, 61). However, the results were contradictory in HSCT and solid organ transplantation (SOT). In SOT, Bruminhent’s team proposed a lower risk of CMV infection in patients with higher CMV-IgG titers (57). Yet, Leonardo *et al.* argued that a higher recipient pre-transplantation CMV-IgG titer was significantly associated with the development of the CMV infection (58). The same result was observed in another project (61). It retrospectively analyzed 309 HSCT patients and found that CMV infection occurred most frequently in the high-titer group. Based on the observation of clinic practices, a higher CMV-IgG titer might indicate humoral immunity against CMV itself in organ transplantation and increased viral load of latently infected CMV in HSCT recipients. The mechanism is still unclear, and more projects should be carried out to identify the role of CMV-specific antibodies in CMV infection.

#### 4.2.2 CD4^+^/CD8^+^ specific T cell response

Until recent years, people start to be aware that the different efficacy in antiviral treatment among recipients is closely related to the CMV-specific T cell immune response. Assessment of CMV-specific T cells may help evaluate the risk of CMV infection, or monitoring of CMV-specific T cells during the onset of CMV infection may help adjust the strategy of antiviral therapy. Thus, attempts have been made to measure CMV-specific-cell-mediated immunity (CMI) in HSCT recipients. In current assays, CMV-CMI is mainly acquired by indirectly quantifying cytokines (TNF α, IFN γ, IL-2, etc.) or directly measuring the number of those CMV-specific T cells.

QuantiFERON, an ELISA-based assay indirectly measures CMV-specific IFN-γ (62). The stimuli in this test include 23 CMV peptide epitopes, which allows it to cover a wide spectrum of CMV subtypes (63). And its testing efficacy has been well illustrated by multiple prospective studies (63–65). Another IFN-γ–based assay, ELISPOT, measures the cytokine of mononuclear cells in peripheral blood following *ex vivo* stimulation of CMV antigens (IE-1 and pp65). Its better concordance with CMV infection in patients with a low CMV-CMI made it a sensitive approach (66, 67). However, it failed to show a difference in the project of Barron *et al.* (68), therefore the efficacy is still debatable. Flow cytometry with intracellular cytokine staining (ICS) can not only measure multiple CMV-specific cellular markers, but also the number of CMV-specific T cells (69). However, it lacks standardization. In addition, clinicians may obtain different results depending on the method selected to measure CMV-specific CMI and large-scale prospective studies are insufficient.

Despite the limitation, CMV-CMI seems to affect the development and prognosis of CMV infection. Cellular immunity mediated by T cells is known to be responsible for controlling CMV replication, and the absence or delayed recovery of CMV-specific T cells causes CMV infection and CMV diseases (70–72). Even though more studies are necessary before their practical applications, monitoring CMV-specific antibodies and CMV-specific cell responses, may help in determining the duration of prophylaxis and treatment, thereby minimizing drug exposure.

## 5 Current therapeutic strategies for CMV infection in HSCT

### 5.1 Preemptive treatment

Preemptive treatment is the most widely used strategy for the prevention of CMV infection after HSCT, which is initiated right after the early detection of CMV infection.

#### 5.1.1 Antiviral agents for treatment

Antiviral agents serve as the backbone for CMV treatment in transplant recipients (73): the first-line anti-CMV medication includes intravenous ganciclovir or oral valganciclovir, while foscarnet and cidofovir are considered second-line agents that are reserved for treatment of resistant and refractory CMV. Antiviral agents have been reported to achieve CMV elimination in up to 70% of cases, which greatly reduces the incidence of CMV disease, one of the most dangerous post-transplant complications, whose current morbidity is nearly10% of HSCT patients (23, 50). However, routine usage of preemptive therapy also shows significant side effects including granulopenia/agranulocytosis (ganciclovir and valganciclovir) and renal dysfunction (foscarnet and cidofovir) (52, 74). Maribavir, a novel antiviral agent for CMV, has been brought up recently. It is a benzimidazole antiviral that inhibits viral replication by inhibiting the viral protein kinase UL97 (64). After successful Phase 1 and 2 trials, maribavir failed to show efficacy in the initial Phase 3 clinical trial (75). Although maribavir is not routinely used as a first-line preemptive treatment, it is recommended as a second-line treatment for resistant CMV infection, because its therapeutic efficacy has been proved in more than half of the refractory and drug-resistant cases in Robin K Avery’s study (76). Recently, drug resistance has been reported with the emergence of the mutations in the UL97 kinase, particularly the virus strains that resist ganciclovir and its pro-drug valganciclovir, since they have been used as first-line drugs in approximately 90% of patients (77). However, resistance can develop with all antiviral drugs used for CMV prophylaxis and therapy. Therefore, the resistance must be considered in patients whose CMV load still increases even though they are on appropriately dosed therapy for 2 weeks.

#### 5.1.2 CMV intravenous immunoglobulin (CMV-IVIG) for treatment

CMV-IVIG functions to prevent CMV from entering the host cells. Besides, this CMV-specific antibody facilitates cytotoxicity, phagocytosis, and complement-mediated cytolysis (78–80). With these capabilities, strategies combining CMV-IVIG and antiviral agents have been proposed for CMV treatment: some found that, for preemptive treatment, this IVIG reduced the high risk of CMV infection from 62% to 36% (81–83). Compared to antiviral agents, CMV-IVIG offers an alternative option for CMV treatment without renal and bone marrow impairment. However, according to the 2017 European Conference on Infections in Leukaemia (ECIL 7) guideline, the addition of CMV-IVIG to second-line or third-line treatment is not recommended, except for the CMV pneumonia (1), for there is still a lack of solid evidence.

#### 5.1.3 Adopted T cell therapy

Since cellular immunity is essential for the control of CMV infection, the use of CMV special cytotoxic lymphocytes (CTLs) becomes an attractive emerging therapy. There are various ways to generate CMV-specific T cells, which involve stimulation of CMV-specific cells by using the viral peptides, protein, lysate, or antigen-presenting cells. Then, CMV-specific T cells can be expanded *in vitro* or by direct infusion into the recipients, thus proliferating under a physiological environment *in vivo* (84). As reported, adoptive CMV special T cells therapy can be useful in controlling resistant and refractory CMV infection. In a prospective multicenter clinical trial, HSCT patients with refractory CMV infection received *ex vivo* CMV-specific T cells, were all detected CMV epitope CMV-specific T cells (85). Another study from a single center examined the CMV-specific T cells expansion of 32 HSCT patients with refractory CMV infection and showed that 27 of 32 (84.4%) patients had a resolution of CMV infection within 4 weeks after adoptive T-cell transfer, and did not experience further viral recurrence (86). However, long-term *in vivo* persistence of transferred CMV-specific T cells required the development of an endogenous CMV-specific T-helper response. Therefore, the limitation restrains these strategies from being popularized in the clinical practice (87). Moreover, current studies that used adoptive special T cells for the treatment of resistant and refractory CMV infection were limited by small numbers and lacked comparison groups in the setting of randomized controlled trials (85, 86, 88, 89). More studies are needed to assess its efficacy.

Though preemptive treatment greatly reduces the risk of CMV infection, several studies have demonstrated that CMV infection after HSCT was associated with increased overall and all-cause mortality, and the risk increases with the viral load (9, 90), independent of the use of preemptive therapy (90, 91). Therefore, prevention of viral replication, rather than surveillance-based preemptive therapy, is considered a more suitable choice for allogenic transplant recipients at high risk of CMV infection.

### 5.2 General prophylaxis

General prophylaxis usually refers to the usage of antiviral agents to prevent CMV infection prior to HSCT. The antiviral agent is administered shortly before transplantation, and continued for at least 3 months (92, 93).

#### 5.2.1 Antiviral agents for prophylaxis

In the 1980s and 1990s, anti-CMV prophylaxis with high-dose acyclovir or valacyclovir was brought up for the first time owing to the efficacy of suppressing the CMV infection (94). For decades, effective agents to control CMV infection were limited to those with significant toxicity, such as ganciclovir, foscarnet, and cidofovir. And ganciclovir is the only drug that has been evaluated as prophylaxis in randomized trials (17, 95, 96). However, it failed to improve the overall survival due to severe neutropenia, as well as secondary bacterial and fungal infections (1, 18, 97). Thus, antiviral prophylaxis has not been considered a routine or common approach for most centers.

Recently, clinical trials on novel antiviral agents for CMV prophylaxis have been studied. Letermovir, a novel antiviral agent that inhibits the viral terminase complex without major toxic effects, is recently approved to decrease CMV infection after HSCT in phase 3, randomized, placebo-controlled trial (98). After the result of this trial, many centers have adopted the use of letermovir prophylaxis to prevent CMV infection after HSCT. The result of this trial has been replicated in the real world too (99, 100). Despite its favorable clinical efficacy, there appeared to be an increased rate of late CMV events after the discontinuation of the letermovir prophylaxis (98). A prospective study of 56 allogenic transplant recipients who received letermovir as prophylaxis, compared to those who received PCR-guide preemptive therapy, showed that letermovir may delay CMV-specific T cell reconstitution, which is possibly related to decreased CMV antigen exposure (101). Moreover, mutations in CMV UL56 terminase leading to letermovir resistance in HSCT recipients have been reported (102), yet further evaluation is still necessary. In addition to the drug resistance, false-positive cases were also reported (103). Letermovir inhibits the terminal phase of CMV replication by targeting the CMV terminase complex. As a result, a very late stage of CMV replication is inhibited and the long DNA concatenator is unable to split into individual viral subunits, producing noninfectious long DNA molecules, which can be detected by the current highly sensitive CMV QNAT method in the blood sample. Therefore, the results of CMV QNAT during letermovir prophylaxis may be misinterpreted.

Another novel antiviral agent, brincidofovir, is a lipid conjugate prodrug of cidofovir, which reduces the nephrotoxic and marrow toxic side effects of cidofovir. A phase 3 randomized controlled trial that compared brincidofovir to placebo for CMV prophylaxis in HSCT patients failed to show a reduction in clinically significant CMV infection at 24 weeks. The agent was also associated with an increased incidence of GVHD (104), and its use was not approved for this indication. In addition, current antiviral agents do not target CMV during latency, but viral replication. This highlights the potential for clinically relevant recurrence of CMV infection following therapy cessation.

#### 5.2.2 CMV-IVIG for prophylaxis

After HSCT, the deficiencies of cellular and humoral immunity give chances to viral infections. Owing to the immune deprivation before immunological reconstitution or persistent immune dysfunction in post-HSCT, several studies started to focus on CMV-IVIG in preventing CMV infection and disease in the past decades. There was evidence from the 1980s to 1990s indicating that prophylactic use of CMV-IVIG was associated with reduced incidence of CMV infection and improved disease condition (105, 106). Following these studies, randomized controlled trials and large meta-analyses refuted the role of CMV-IVIG in preventing CMV infection, for it found no significant effect on CMV infections or overall mortality (107, 108). As a result, CMV-IVIG was not included in the updated guidelines for CMV prevention after HSCT. However, with the development of HSCT technology including reduced-intensity conditioning and the elder population of patients receiving HSCT, present clinical practice in HSCT may differ from the previous one (107). In addition, recent technical development of immunoglobulin production has improved intact CMV-IVIG preparations with normal half-lives, effector functions, higher pathogen safety, and fewer coagulating factors (109). Taken together, the previously available data may not be adequate to inform current HSCT practices. Accordingly, several recent clinical trials have suggested that prophylactic CMV-IVIG showed a significant reduction in CMV infection (83, 110, 111)(**Table 2**). Given the well-established mechanisms and its safety profile as well as less toxicity, CMV-IVIG has a promising future as prophylaxis in selective patients who are susceptible to CMV infection. Likewise, further randomized controlled trials also are needed.

**Table 2:** Trials of CMV-IVIG prophylaxis for CMV infection in the past five years.

							CMV infection		
References	Cohort	Eligibility criteria	Cases	CMV-IVIG Dose	Regime	Control group	Treatment group (%)	Control group (%)	Follow-up
Gal et al. (2017) (110)	Israelite	first-time allo-HSCT HLA-identical sibling	109	500mg/kg	Day -7 to 0, then *qw* till +100d	No treatment	10 (12.2%)	11 (40.7%)	1 year
Danniel et al. (2019) (111)	American	CMV D+/R− allo-HSCT	53	200 mg/kg	on Day -8, -6, 0, 7, 14, 21, 28, 42, 56, 70	No treatment	7 (29.2%)	12 (41.4%)	100 days
Michele et al. (2019) (83)	Italian	allo-HSCT received at least one dose of CMV-IVIG	92	50UI/kg	Different schedules	No treatment	0 (0%)	59 (75.6%)	100 days

#### 5.2.3 Adopted T-cell therapy

CMV CTLs immunotherapy has been used more frequently in HSCT. Some centers have investigated its use as a prophylactic measure to prevent CMV infection in HSCT recipients, and showed that adoptive T cell therapy is beneficial to prevent CMV infection (112, 113). In one study, prophylactic adoptive transfer of *ex vivo* CTLs was given to nine R^+^ HSCT recipients. Two R^+^ developed CMV viremia, while none of them required antiviral treatment and were cured (112). Another study showed that infusions of low-dose donor memory T-lymphocytes may lead to the expansion of CMV CTLs, and therefore prevent CMV infection in HSCT transplantation (113).

However, these studies were limited by insufficient sample sizes and the lack of comparison groups in the setting of randomized controlled trials. Before clinical practice, additional large-scale studies are necessary to further evaluate its efficacy and side effects.

#### 5.2.4 CMV vaccines

The development of an effective and safe vaccine against CMV remains an important medical priority. Few vaccine trials for prophylaxis of CMV infection are currently underway (114, 115). ASP0113, the DNA vaccine, is the most studied in HSCT recipients. ASP0113 contains two types of plasmids encoding the CMV antigens tegument pp65 and gB: the former is a major structural protein of CMV, and it is also a dominant CMV antigen recognized by CD4^+^ and CD8^+^ T cells (116); the latter is a surface protein that is neutralized by antibodies (117). Therefore after being vaccinated, pp65 induces T cell-mediated responses, while gB causes the release of viral-specific antibodies, representing the activation of host cellular and humoral immunity respectively (118). The prophylactic effectiveness of this CMV vaccine has been proved in a phase 2 study (115), yet its phase 3 study is being still carried on. Even though CMV vaccine development is promising, it appears that the translation to clinical practice may still take years.

### 5.3 CMV disease treatment during early CMV infection

Since the prevalence of therapeutic strategies against CMV infection, the incidence of CMV disease in the early stages of HSCT has been reduced, and the current morbidity rate is nearly 3% (119). The most common CMV disease has switched from CMV pneumonia to CMV gastrointestinal disease. Despite the rareness, the mortality of CMV pneumonia remains high. Once the diagnosis is confirmed, a combination of intravenous ganciclovir and high-dose IVIG is standardly recommended (120–122). However, given the non-randomized setting of these studies, the efficacy of IVIG is still debatable. For other CMV diseases, ganciclovir is the first-line choice. When the toxic effects or drug resistance restrain the usage of ganciclovir, foscarnet and cidofovir can be chosen as substitutes (1). Maribavir is recommended as a second-line treatment for resistant CMV infection (76).

## 6 Summary

CMV is a highly prevalent, opportunistic pathogen that continues to cause CMV infection and CMV disease, whose increased morbidity and mortality are considered as a big challenge for HSCT. To better prevent and control its infection, further understanding of the conditions that enable its susceptibility to infection is required. Future strategies of diagnosis might involve plasmatic metabolomics profiling to predict the emergence of CMV infection. Though current treatment against CMV has been summarized (**Table 3**), novel therapies for CMV infection are actively being pursued and these will benefit the HSCT recipients in the near future.

**Table 3:** Preemptive and prophylaxis treatment for CMV infection.

		Preemptive	Prophylaxis
Ganciclovir	Dose	5mg/kg *iv q12h*	Day 0-5 5mg/kg *iv q12h;* Day 6-100 5mg/kg *iv qd*
	CMV infection/disease	NA/4.8%-14% (95, 96)	3% (91)/2.7% (95)
	Side effect	11% (severe neutropenia) (96)	30% (severe neutropenia) (92)
Valganciclovir	Dose	900mg *po bid*	900mg *po qd*
	CMV infection/disease	40%/NA (123)	28%/2% (94)
	Side effect	40% (hematological toxicity) (123)	7-8% (Nausea and Vomiting) (94)
Foscarnet	Dose	60mg/kg *iv q8h* or 90mg/kg *q12h*	NA
	CMV infection/disease	NA/4.5% (96)	NA
	Side effect	5% (impaired renal function), 4% (severe neutropenia) (96)	NA
Letermovir	Dose	NA	480mg *po qd*(240mg if with CsA)
	CMV infection/disease	NA	16%/1.5% (98)
	Side effect		18.5% Vomiting and 14.5% edema (98)
CMV-IVIG	Dose	100mg/kg, within 3 days after CMVemia	different schedules (83, 110, 111)
	CMV infection/disease	NA/4.8% (92)	0-29.2% (83, 110, 111)/NA
	Side effect	Infusion reactions	Infusion reactions
Adopted T cell therapy	Graft	*ex vivo* CMV CTLs	*ex vivo* CMV CTLs or donor memory T-lymphocytes
	outcome	84.4%-100% responded (83, 84)	64.5%-66.7% responded (112, 113)
	Side effect	Infusion reactions	No significant adverse event

## Author contributions

BL and XL offered direction and helped on the whole project. JC, KZ, and YS drafted the manuscript. XZ and RW modified the manuscript. BL review the manuscript and made significant revisions on the manuscript. All authors contributed to the article and approved the submitted version.

## Funding

This work was supported by the National Natural Science Foundation of China (81700149), Guangdong Basic and Applied Basic Research Foundation (No. 2020A1515010199), and the Fundamental Research Funds for the Central Universities (No. 20ykpy30).

## Conflict of interest

The authors declare that the research was conducted in the absence of any commercial or financial relationships that could be construed as a potential conflict of interest.

## Publisher’s note

All claims expressed in this article are solely those of the authors and do not necessarily represent those of their affiliated organizations, or those of the publisher, the editors and the reviewers. Any product that may be evaluated in this article, or claim that may be made by its manufacturer, is not guaranteed or endorsed by the publisher.
